# Identification and validation of a costimulatory molecule-related signature to predict the prognosis for uveal melanoma patients

**DOI:** 10.1038/s41598-024-59827-5

**Published:** 2024-04-21

**Authors:** Minyao Zhao, Yue Yu, Zhengyu Song

**Affiliations:** Department of Ophthalmology, Shanghai Shuguang Hospital, Shanghai University of Traditional Chinese Medicine, Shanghai, 201203 China

**Keywords:** Uveal melanoma, Costimulatory molecular, Biomarker, Prognosis signature, Tumor immune microenvironment, Databases, Cancer, Computational biology and bioinformatics, Immunology

## Abstract

Uveal melanoma (UVM) is the most common primary tumor in adult human eyes. Costimulatory molecules (CMs) are important in maintaining T cell biological functions and regulating immune responses. To investigate the role of CMs in UVM and exploit prognostic signature by bioinformatics analysis. This study aimed to identify and validate a CMs associated signature and investigate its role in the progression and prognosis of UVM. The expression profile data of training cohort and validation cohort were downloaded from The Cancer Genome Atlas (TCGA) dataset and the Gene Expression Omnibus (GEO) dataset. 60 CM genes were identified, and 34 genes were associated with prognosis by univariate Cox regression. A prognostic signature was established with six CM genes. Further, high- and low-risk groups were divided by the median, and Kaplan–Meier (K-M) curves indicated that high-risk patients presented a poorer prognosis. We analyzed the correlation of gender, age, stage, and risk score on prognosis by univariate and multivariate regression analysis. We found that risk score was the only risk factor for prognosis. Through the integration of the tumor immune microenvironment (TIME), it was found that the high-risk group presented more immune cell infiltration and expression of immune checkpoints and obtained higher immune scores. Enrichment analysis of the biological functions of the two groups revealed that the differential parts were mainly related to cell–cell adhesion, regulation of T-cell activation, and cytokine–cytokine receptor interaction. No differences in tumor mutation burden (TMB) were found between the two groups. *GNA11* and *BAP1* have higher mutation frequencies in high-risk patients. Finally, based on the Genomics of Drug Sensitivity in Cancer 2 (GDSC2) dataset, drug sensitivity analysis found that high-risk patients may be potential beneficiaries of the treatment of crizotinib or temozolomide. Taken together, our CM-related prognostic signature is a reliable biomarker that may provide ideas for future treatments for the disease.

## Introduction

Uveal melanoma (UVM) is the most common primary malignant tumor in the eye, with 90% of cases found in the choroid, 6% in the ciliary body, and 4% in the iris^1^. There is no gender preference for UVM onset, but it is more common in middle-aged Caucasians with a median age of 58^2^. The clinical features and biology of UVM are distinguished from cutaneous melanoma^3^. It is widely accepted that the development of UVM is a complex process involving multifactorial, multistage, and multigenic mutation accumulation and interaction^4,5^. Recurrent oncogenic mutations and chromosomal copy number aberrations contribute to the development of UVM, and in almost all cases, mutually exclusive mutations are present at an early stage^6,7^.

Treatment options for primary UVM includes ophthalmectomy and radiation therapy^1,7,8^. Despite effective treatments for the primary lesion, distant metastases still occur in 40–50% of patients, with liver metastases accounting for most cases^9,10^. Due to the limited treatment options for metastatic uveal melanoma (mUVM), the median survival of patients after metastasis is only 12 months^11^. Chemotherapy did not improve the patients’ prognosis because the mUVM was highly resistant to systemic cytotoxic chemotherapy^2,12,13^. Although immune checkpoint inhibitors (ICIs) effectively treat cutaneous melanoma, anti-CTLA4 and anti-PD-1 therapy have limited effects in mUVM^14–16^. Tumor-infiltrating lymphocytes (TILs) and tumor mutational burden (TMB) together affect the efficacy of ICIs, and studies have found that tumor-driven immune exclusion may be the cause of immunotherapeutic resistance^17–19^.

Costimulatory molecules (CMs) play a critical role in the biological function of T-cells. The immune response is a complex network, and T cells require CMs for differentiation, proliferation, and survival to generate adaptive immunity^20^. T-cell activation requires two signals. In the case of CD4^+^ T cell activation, the major histocompatibility complex (MHC) peptide complex delivered by the Antigen-presenting cells (APC) is recognized and bound by the T cell receptor^20–22^. At this point, T cells need CMs to amplify the first signal and generate a population of effector T cells and memory T cells. Without the involvement of CMs, even if T cells have recognized the antigen, they will remain in a state of inactivity. Therefore, co-signaling molecules are key in regulating T cell activation, subpopulation differentiation, effector function, and survival. CMs can be categorized into the B7-CD28 family, the TNF family, and the most important known ones^20^.

With advances in tumor immunology, immunotherapy has been applied in the treatment of multiple solid tumors^23^. Immune-checkpoint inhibitors combined with anti-angiogenic therapies increase the infiltration of immune effector cells into the tumor to achieve the anti-tumor effect. The efficacy of tumor immunotherapy largely depends on the tumor immune microenvironment (TIME)^24^. The emergence of ICIs has left an indelible imprint on the immunotherapy of tumors. ICIs are targeted therapies against CMs within the TIME^25,26^. The diverse expression of CMs is involved in TIME construction, immune tolerance, and immune escape, which correlate with patient prognosis and treatment response. CMs are potential prognostic signatures^27–30^.

Due to the specificity of the lesion, next-generation sequencing data for UVM is limited. However, valuable information for disease diagnosis and prognosis can still be obtained by mining the limited data. This study systematically examined the relationship between the prognostic CM gene expression and clinical characteristics in patients with UVM. In addition, through least absolute shrinkage and selection operator (LASSO) regression, we established a CM-related risk model to divide patients into high- and low-risk groups. The predictive model was validated in The Cancer Genome Atlas (TCGA) and Gene Expression Omnibus (GEO) databases, indicating excellent predictive efficiency. Subsequently, univariate and multiple Cox regression analyses demonstrated that risk score was an independent risk factor for prognosis. Then, we integrated other clinical characteristics and risk scores to construct a nomogram to predict the prognosis. At last, we compared the differences in clinicopathological features, immune cell infiltration, biological function, and somatic mutations between high- and low-risk groups.

## Materials and methods

### Database download and processing

The RNA sequencing data and clinical information of UVM patients were obtained from the TCGA database (https://portal.gdc.cancer.gov/), which included 80 patients. In addition, the validation dataset was derived from the GEO dataset (https://www.ncbi.nlm.nih.gov/), GSE22138, containing 63 patients and more than 36 months of follow-up records^31^.

### Identification of costimulatory molecules

Based on previous studies^29,30^, 60 CM genes were obtained, and the CMs can be categorized into two superfamilies: the *B7*-*CD28* family and the TNF family. The *B7*-*CD28* family includes eight *B7* family genes (*CD274*, *CD276*, *CD80*, *CD86*, *HHLA2*, *ICOSLG*, *PDCD1LG2*, *VTCN1*) and five CD28 family genes (*CD28*, *CTLA4*, *ICOS*, *PDCD1*, *TMIGD2*). The TNF family consists of 18 *TNSF* and 29 *TNFRSF* family genes.

### Construction and validation of the costimulatory molecule‑related prognostic signature

The TCGA-UVM patients were used as a training set, and the CM genes were extracted and screened for prognosis-related genes by univariate Cox regression analysis (*P* < 0.05). Prognosis-related genes were screened, and prognosis models were constructed through LASSO regression using the R-package “glmnet”^32^. Lasso algorithm is a linear regression algorithm, that can realize feature selection and dimensionality reduction, and has a good effect on high-dimensional data such as transcriptome data. The regularization of LASSO was determined by the minimum parameter (λ). Risk scores were calculated by multiplying the expression level of the genes and the multivariate Cox proportional hazards coefficient. The detailed formula was ${\text{RiskScore }} = {\text{ Exp1}} \times \beta {\text{1 + Exp2 }} \times \beta {\text{2 + Exp3 }} \times \beta {\text{3 + Expi}} \times \, \beta {\text{i}}$. Patients were divided into high- and low-risk groups based on the median risk score. We included overall survival (OS), progression-free interval (PFI), and occurrence of metastasis as outcomes. Kaplan–Meier (K-M) curves compared the prognostic differences between the two groups. The time-dependent receiver operating characteristic curve (timeROC) and area under curve (AUC) was calculated by the R package “timeROC” to assess the predictive efficacy of prognostic signature over time. Finally, we validated the prognostic signatures with an external dataset^31^.

### Consensus clustering analysis of prognosis‑related costimulatory molecule genes

To further explore the prognostic value of CM genes, we performed a consensus clustering analysis of prognostic-related genes with the R-package “ConsensusClusterPlus”. The package supports running consensus clustering at different numbers of clusters (k values) and can automatically select the optimal number of clusters. The distribution of patients in different clustered subgroups was assessed by principal component analysis (PCA). K-M curves compared prognostic differences between clusters.

### Assessment of immune cell infiltration

Based on the RNA transcriptome data, the abundance of 22 immune cell subpopulations in each sample was calculated with the R-package “CIBERSORT”^33^ and “ssGSEA”. The level of immune cell infiltration in the tumor tissue was assessed by the R-package “ESTIMATE”^34^, which includes the stromal score, the immune score, and the ESTIMATE score. The stromal score and immune score are designed to assess the presence of stromal cell and immune cell infiltration in tumor tissue, respectively. The ESTIMATE score had a significant negative correlation with tumor purity in samples. Pearson correlation analysis was performed to compare the correlation analysis between the expression of individual prognostic genes and the degree of infiltration of individual immune cells, as well as the correlation analysis between risk score and the degree of infiltration of individual immune cells.

### Identification of differentially expressed genes

Patients were divided into high- and low-risk groups based on the median of the risk score and differentially expressed genes (DEGs) were compared between patients based on the criteria of *P* < 0.05 and |log_2_(Fold Change)|> 1.

### Functional enrichment analysis based on DEGs

We selected DEGs in high- and low-risk groups for Gene Ontology (GO) analysis and Kyoto Encyclopedia of Genes and Genomes (KEGG, https://www.kegg.jp/kegg/kegg1.html)^35^ enrichment analysis to explore the biological pathways associated with the prognostic signature. The above comment was realized by the Metascape platform (https://www.metascape.org/). The R-package “clusterProfiler” was utilized to perform Gene Set Enrichment Analysis (GSEA) in the gene set (c5.all.v7.5.1.entrez.gmt) to discover potential biological processes through clustering^36^.

### Mutation analysis

Somatic mutation status data for UVM samples were downloaded from the TCGA data portal (https://portal.gdc.cancer.gov/repository). Mutation data were screened using the R-package “maftools” and compared between high- and low-risk patients^37^. Maftools provides a wide range of analysis and visualization modules commonly used in cancer genomic research, including cancer driver genes identification, pathway, signature, enrichment, and correlation analyses.

### Construction of a nomogram

A nomogram was constructed with the R package “rms” to predict the survival rate of UVM patients at 1, 3, and 5 years based on the patient’s clinical information and risk scores.

### Analysis of drug sensitivity

Based on the Genomics of Drug Sensitivity in Cancer 2 (GDSC2) dataset (https://www.cancerrxgene.org/), we compared drug sensitivity differences among 198 chemotherapeutic agents in high- and low-risk groups with the R package “oncoPredict”^38^. The “oncoPredict” algorithm utilized the expression matrix of GDSC2 and drug response information as training data to calculate the half maximal inhibitory concentration (IC50) value for each drug based on the transcriptome data of the samples provided by the investigators^39^.

### Statistical analyses

We used t-tests for measurement data and chi-square tests for count data for two variables. Univariate and multivariate Cox regression analyses were used to assess the predictive value of each risk factor. The predictive efficacy of prognostic signatures was evaluated with the K-M curve, ROC, and timeROC. Pearson correlation analysis was used to assess the correlation between two variables. The procedures involved in this study were done on R software (version 4.2.1) and SPSS software (version 25.0), and *P* < 0.05 was considered statistically significant.

## Results

### Patient characteristics

The 80 patients in the discovery cohort were obtained from the TCGA database. The dataset provides basic clinical information about the patients, which includes age, gender, survival status, tumor stage, metastasis (TNM) stage, and treatments. Table 1 shows a selection of the patient’s clinical characteristics.

**Table 1:** Detailed clinical and pathological information of TCGA-UVM patients.

Variables	TCGA cohort (n = 80)	Risk score^a^		*P*-value
		High risk	Low risk	
Gender				0.652
Female	35	16	19	
Male	45	24	21	
Age (mean ± SD, years)	61.65 ± 13.95	64.80 ± 12.05	58.50 ± 13.02	0.823
≤ 60 years	40	19	21	
> 60 years	40	21	19	
Stage^b^				0.035*
II	36	14	22	
III	40	22	18	
IV	4	4	0	
Pathological T stage^c^				0.086
T2	4	1	3	
T3	36	14	22	
T4	38	23	15	
NA	2	2	0	
Clinical subtype				< 0.001*
Monosomy 3	42	37	5	
Disomy 3	38	3	35	

^a^Risk scores were calculated by multiplying the expression level of the genes and the multivariate Cox proportional hazards coefficient. Patients were divided into high- and low-risk groups based on the median risk score.

^b^Samples were graded with reference to the AJCC clinical stage.

^c^Samples were graded with reference to the AJCC primary tumor (T).

*Font bold indicates statistical significance (p < 0.05).

### Identification of costimulatory molecular genes valuable for UVM prognosis

**Figure 1:**
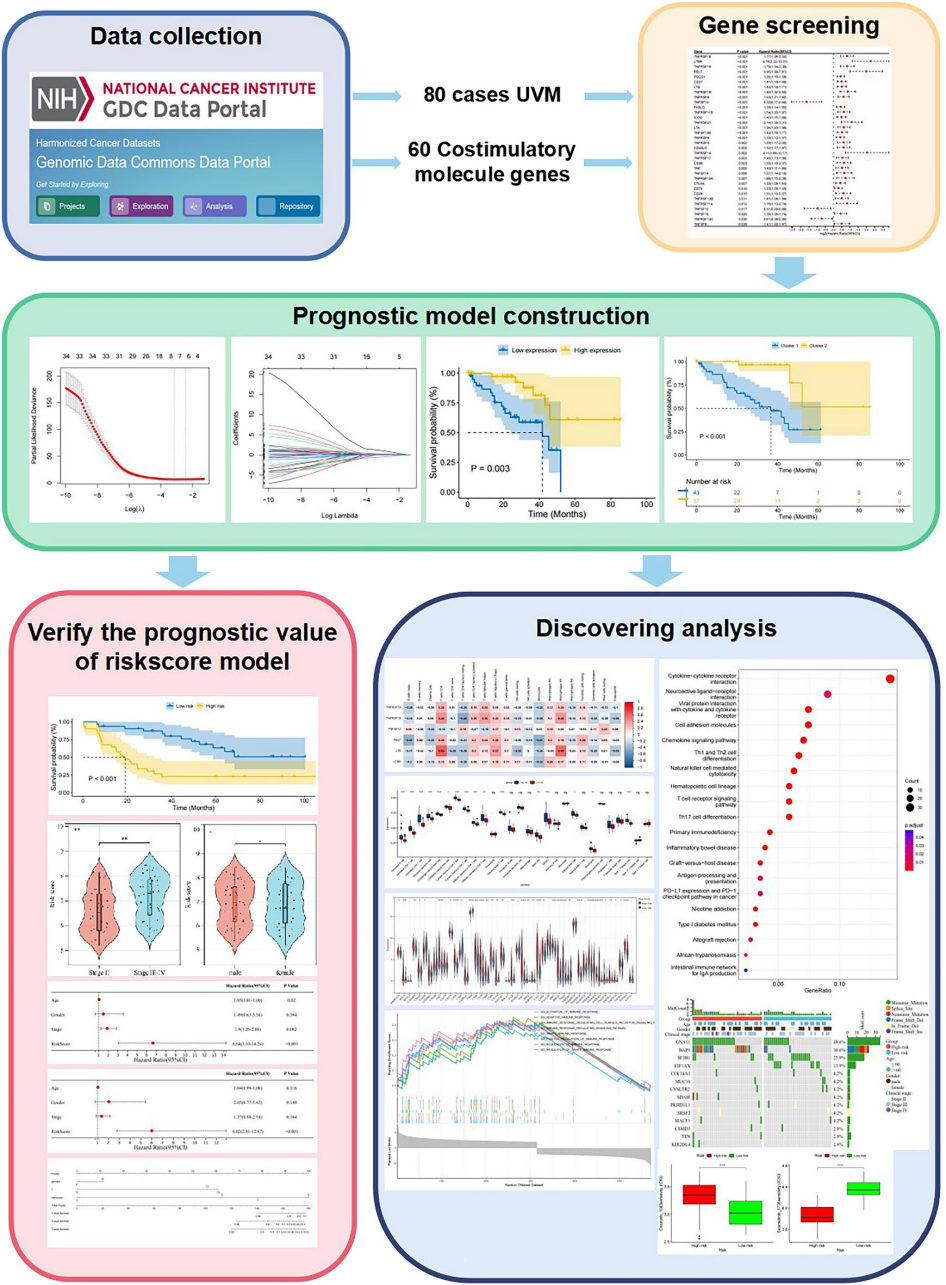
The flowchart of the study.

**Figure 2:**
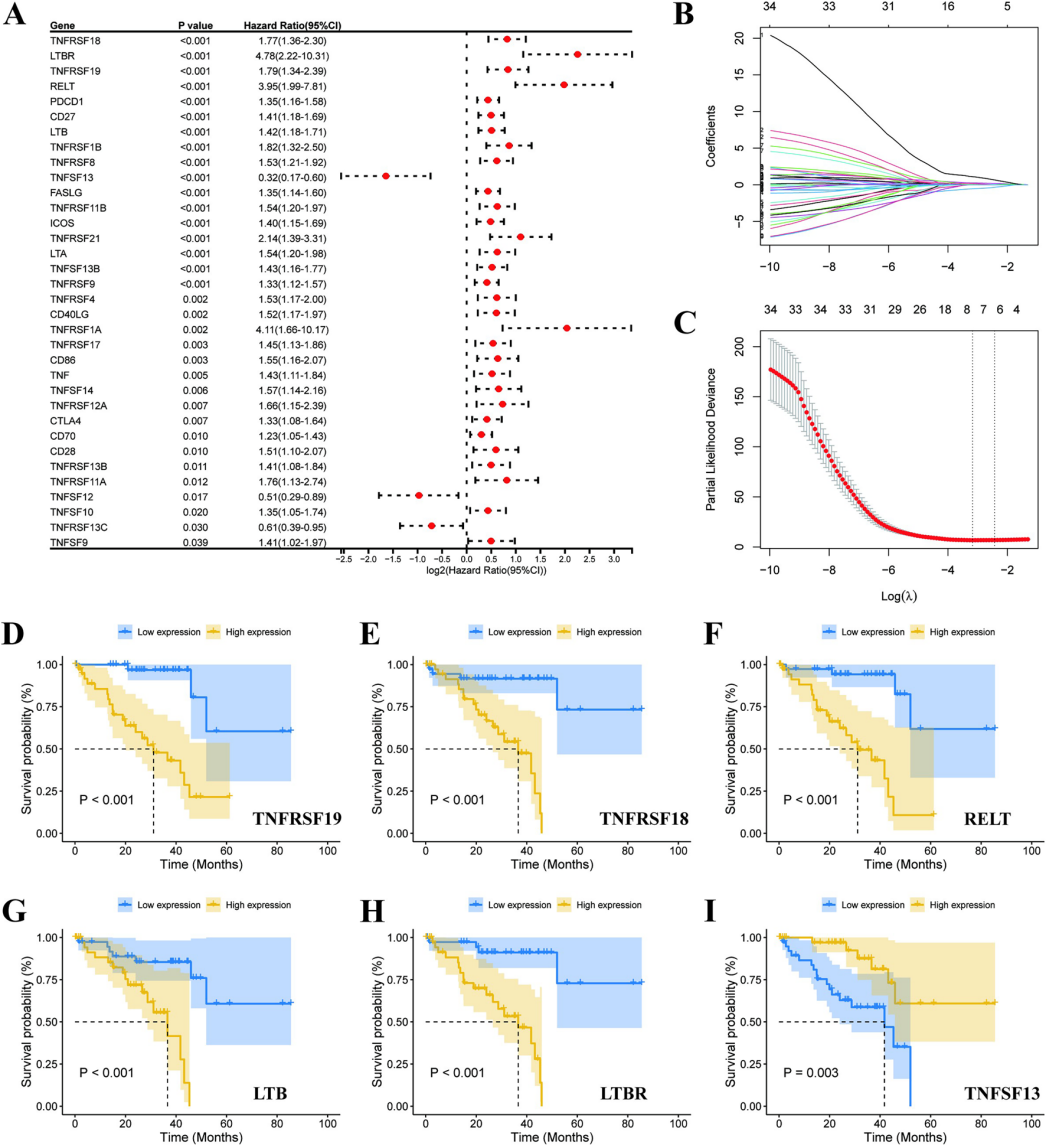
Identification of costimulatory molecular genes associated with UVM prognosis. (**A**) 34 CM genes were identified through univariate Cox regression analysis. (**B**) 10-time cross-validation for tuning parameter selection in the Lasso model. (**C**) LASSO regression of the 34 OS-related genes. The Kaplan–Meier curves of TNFRSF19 (**D**), TNFRSF18 (**E**), LTBR (**F**), RELT (**G**), LTB (**H**), and TNFSF13 (**I**) from the TCGA dataset.

The workflow of this study is demonstrated in Fig. 1. TCGA-UVM database provided 80 cases, and we extracted expression data for 60 CM genes from the transcriptomic data. 34 genes associated with prognosis were obtained using univariate Cox regression analysis with OS as survival time (Fig. 2A, Supplementary Table 1). Further filtering obtained six prognosis-related CM genes by LASSO regression analysis (Fig. 2B, C). K-M curves demonstrated additional prognostic values of each candidate gene. The high expression of *TNFRSF19*, *TNFRSF18*, *RELT*, *LTB*, and *LTBR* was associated with a poor prognosis in UVM patients (Fig. 2D-H). In contrast, the high expression of *TNFSF13* was associated with a better prognosis (Fig. 2I).

### Identification of UVM subgroups through consensus clustering

**Figure 3:**
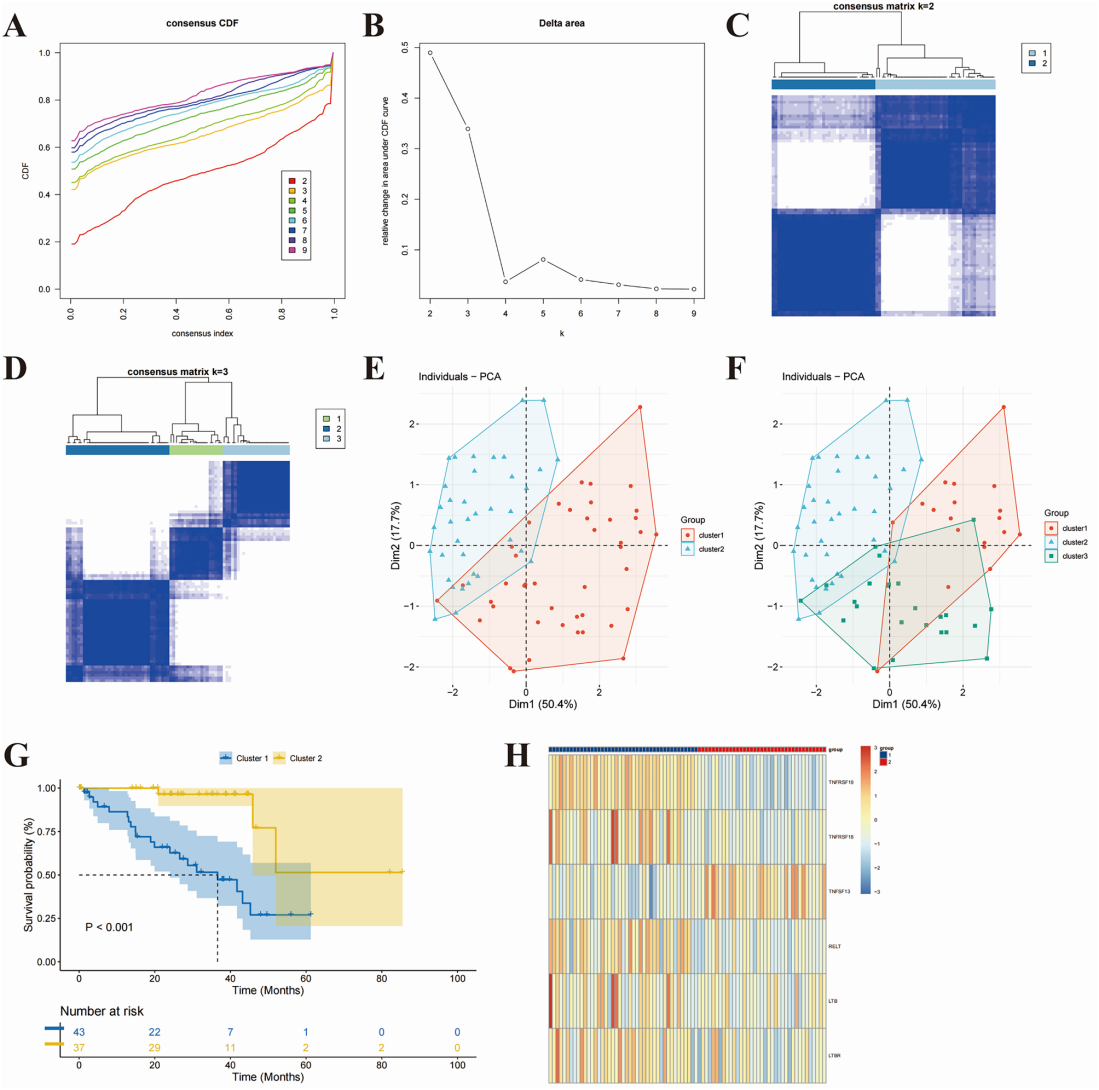
Consensus clustering of costimulatory molecule-related prognostic genes. Clustering analysis based on the expression profile of six CM genes (**A**,**B**). TCGA UVM cohorts were grouped into two (**C**) and three (**D**) clusters according to the consensus clustering matrix. The optimal value for consensus clustering was observed to be k = 2 by principal component analysis (PCA) (**E**,**F**). (**G**) Overall survival demonstrated a poorer prognosis for UVM patients in the cluster 1 cohort compared to cluster 2. (**H**) The heat map of two cohorts along with the expression of CM genes.

To further explore the prognostic value of CMs, we divided UVM patients into clusters by consensus clustering based on transcriptome-wide data. The number of optimal clusters was determined by the cumulative distribution function (CDF) (Fig. 3A). Using the CDF delta area curve, we found that clustering was most stable when the dataset was divided into three clusters (Fig. 3B). From the clustering results, the clusters are relatively stable for either *k* = 2 or 3 (Fig. 3C, D). Further, we verify the reliability of clustering by PCA. Interestingly, cluster 1 is the same group of patients when *k* = 2 and *k* = 3 (Fig. 3E, F). Through the K-M curve, we found that cluster 1 had worse overall survival compared to cluster 2 (*P* < 0.001) (Fig. 3G), suggesting that cluster 1 is a specific subgroup with a poor prognosis. By heat map analysis, costimulatory genes (*TNFRSF19*, *TNFRSF18*, *RELT*, *LTB*, *LTBR*) associated with poor prognosis were enriched in cluster 1 (Fig. 3H).

### Construction and validation of a prognostic model based on six costimulatory molecule genes

The risk score for the prognosis of UVM patients was calculated using the expression levels of the six CM genes multiplied by the coefficient of multivariate Cox proportional hazards. The detailed calculation formula is as follows:

$$RiskScore = \left( {0.19010889 \times TNFRSF19} \right) + \left( {0.02747141 \times TNFRSF18} \right) + \left( { - 0.34006139 \times TNFSF13} \right) + \left( {0.03741758 \, \times RELT} \right) + \left( {0.03364384 \times LTB} \right) + \left( {0.66089535 \times LTBR} \right)$$

**Figure 4:**
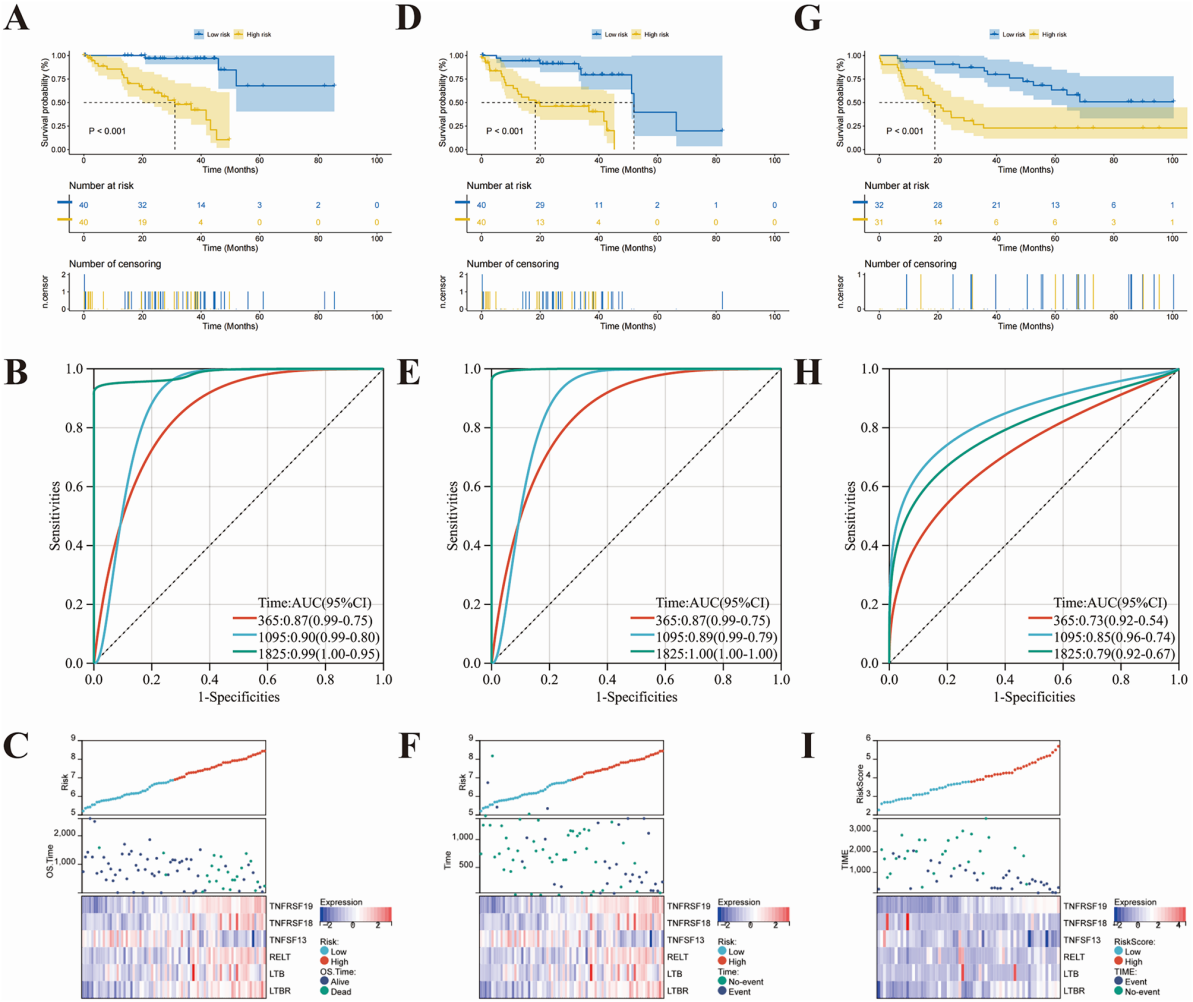
Evaluating the predictive efficacy of the risk score model. Kaplan–Meier curves indicated the difference between high- and low-risk group overall survival (**A**) and progression free interval (**D**) in training cohort. (**G**) Progression free survival analysis for high- and low-risk patients in validate cohort. The ROC curve of measuring the predictive value in training cohort (**B**,**E**) and validate cohort (**H**). (**C**,**F**,**I**) Scatterplot and heatmap demonstrating the distribution of high- and low-risk groups and the expression levels of prognosis-related genes.

The median risk score classified patients into high- and low-risk groups. K-M curves suggested a poorer OS in patients in the high-risk group in the TCGA dataset (*P* < 0.001) (Fig. 4A). A ROC curve was used to demonstrate the ability to recognize the prognosis of the disease. The AUC values for ROC curves of 1, 3, and 5 years were 0.87, 0.90, and 0.99 (Fig. 4B, C). Further, we utilized PFI as a prognostic outcome for the training dataset and found that our risk model could better predict the incidence of short-term and long-term progression in patients (Fig. 4D). The AUC values for ROC curves were 0.87 at 1 year, 0.89 at 3 years, and 1.00 at 5 years (Fig. 4E, F). UVM is highly susceptible to distant metastasis through the bloodstream, leading to poor patient prognoses. Therefore, we chose the GSE22138 dataset, which included 63 patients and at least 36 months of follow-up (Supplementary Table 2). K-M curve indicates earlier metastasis in high-risk patients (*P* < 0.001) (Fig. 4G). The AUC values for ROC curves of 1, 3, and 5 years were 0.73, 0.85, and 0.79, respectively (Fig. 4H, I). The results above demonstrate the high sensitivity and specificity of our prognostic signature.

Association between the prognostic signature and clinical characteristics.

**Figure 5:**
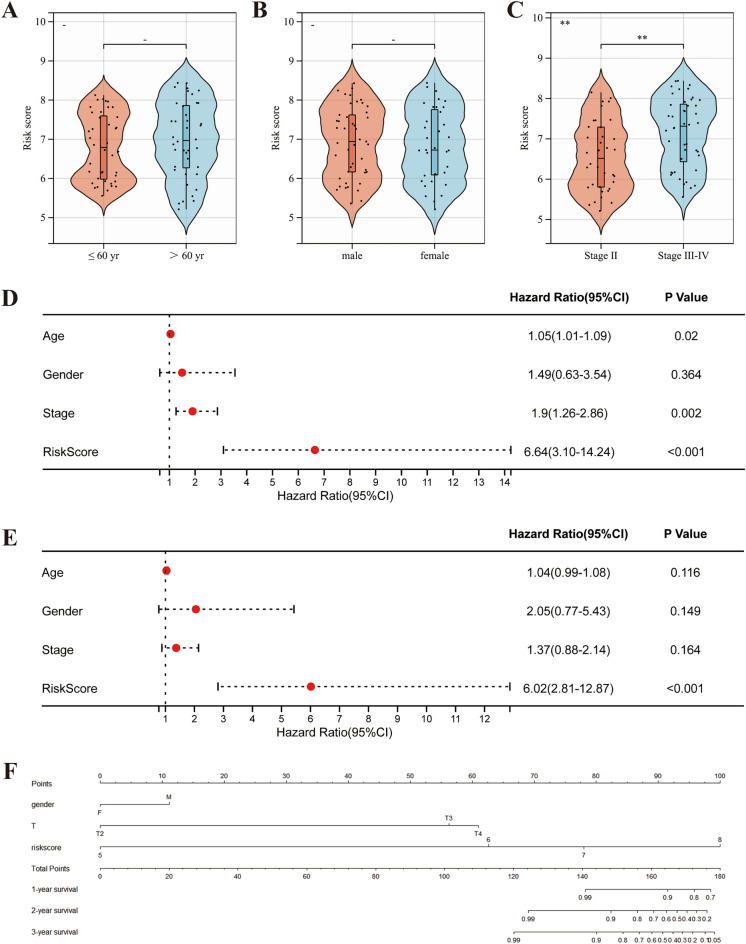
The risk score correlated with clinicopathological features and in UVM. Risk scores were independent of age (**A**) and sex (**B**), but higher in patients with progressive tumors (Stage III, IV) (**C**). Univariate (**D**) and multivariate (**E**) Cox regression analysis of the relationship between each risk factor and prognosis. (**F**) Construction of predictive nomogram to predict the 1-, 2- and 3 year overall survival of UVM patients. *P < 0.05; **P < 0.01; ***P < 0.001.

As the above results demonstrate that our risk signature is a good prognostic model, we compared the variation in risk scores between different clinical subgroups. In clinical characteristics, advanced age and gender did not impact risk scores (Fig. 5A, B). Patients with progressive disease tended to obtain higher scores (Fig. 5C). Further, through univariate Cox regression analysis, age, stage, and risk score were found to be risk factors for UVM patients’prognosis (Fig. 5D). Multivariate Cox regression analysis revealed that risk score (HR = 6.02, 95% CI 2.81–12.87) was the independent risk factor for prognosis (Fig. 5E). Finally, we integrated multiple factors influencing patient prognosis and constructed a nomogram (Fig. 5F).

### Correlation between prognosis signature and immune microenvironment

**Figure 6:**
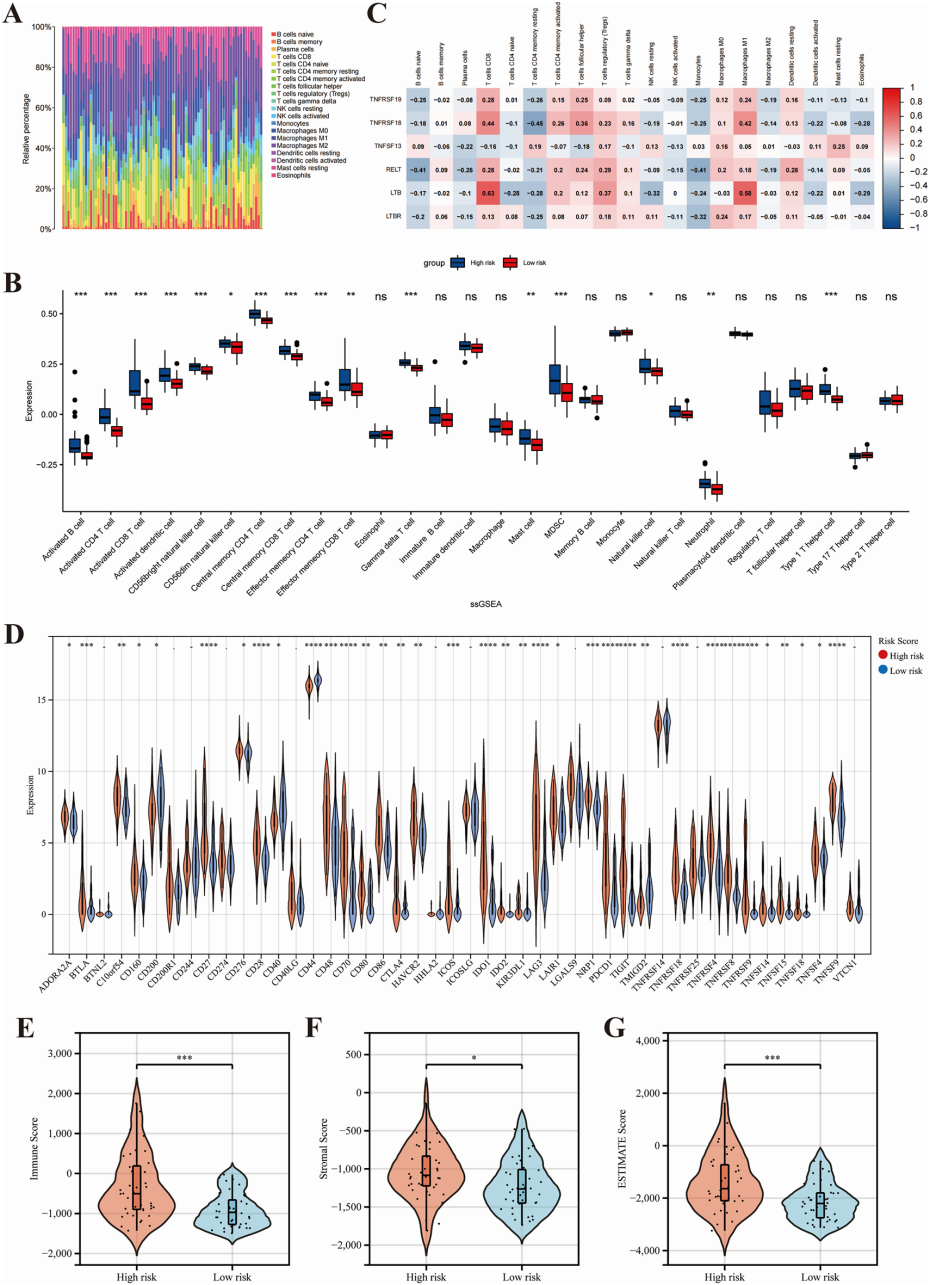
The risk score correlated with immune cell infiltration. (**A**) The bar chart demonstrated the immune cell infiltration in different patients. (**B**) The heat map illustrates the correlation of CM-related prognostic genes with immune cell infiltration. (**C**) The infiltrating levels of 28 immune cell types in high/low-risk groups in UVM. (**D**) The expression of immune checkpoints in UVM. The stromal scores (**E**), immune score (**F**) and estimate scores (**G**) differed between the high- and low-risk patients. *P < 0.05; **P < 0.01; ***P < 0.001.

**Figure 7:**
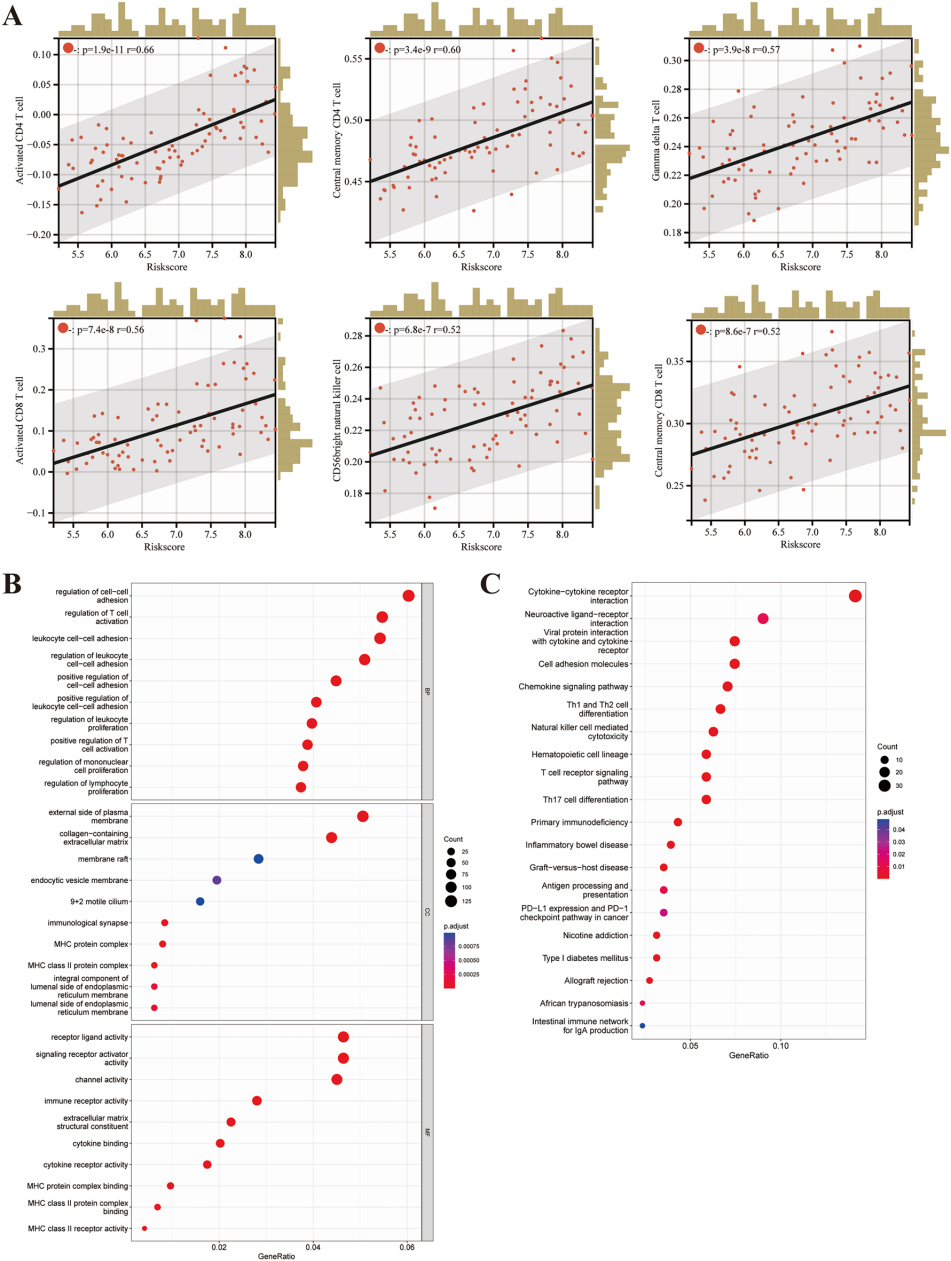
Enrichment analysis of differentially expressed genes (DEGs) between high- and low-risk patients. (**A**) High infiltration of immune cells positively correlated with high risk scores, including activated CD4 T cell, central memory CD4 T cell, gamma delta T cell, activated CD8 T cell, CD56bright natural killer cell, and central memory CD8 T cell. The representative results of GO enrichment (**B**) and KEGG pathways (https://www.kegg.jp/kegg/kegg1.html) analysis of DEGs (**C**). *BP* biological process, *CC* cellular component, *MF* molecular function.

The stacked bar chart displayed the infiltration of 22 types of immune cells in each sample in which macrophages and CD4^+^ T cells were the predominant infiltrating immune cells (Fig. 6A). Further analysis by correlation of prognostic signature genes and immune cell infiltration showed that the high expression of LTB and TNFRSF18 were positively correlated with infiltration of CD8+ T cells and macrophages M1 (Fig. 6B). The bar graph demonstrated that high-risk patients had more immune cell infiltration within the tumor tissue, including activated B cells, activated CD4 T cells, activated CD8 T cells, activated dendritic cells, and so on (Fig. 6C). It suggested that high-risk scores could identify hot tumors. High expression of immune checkpoint genes in tumors indicates that patients are more likely to benefit from treatment with immune checkpoint inhibitors. More immune checkpoint genes were highly expressed in high-risk patients, including *ADORA2A*, *BTLA*, *CD16*, *CD27*, *CD276*, *CD48*, *CD70*, *CD80*, *CD86*, *CTLA4*, and so on (Fig. 6D). Most of the immune checkpoints are part of the CMs. Higher immune scores, stromal scores, and ESTIMATE scores were found in high-risk patients (Fig. 6E–G). The Pearson correlation analysis also revealed that the high-risk score was positively correlated with the infiltration of a variety of immune cells, including activated CD4 T cells, central memory CD4 T cells, gamma delta T cells, activated CD8 T cells, CD56bright natural killer cells, and central memory CD8 T cells (Fig. 7A). The results above indicated that high-risk patients were often associated with a higher percentage of immune cell infiltration.

### Identification of the biological pathways associated with prognostic signature

**Figure 8:**
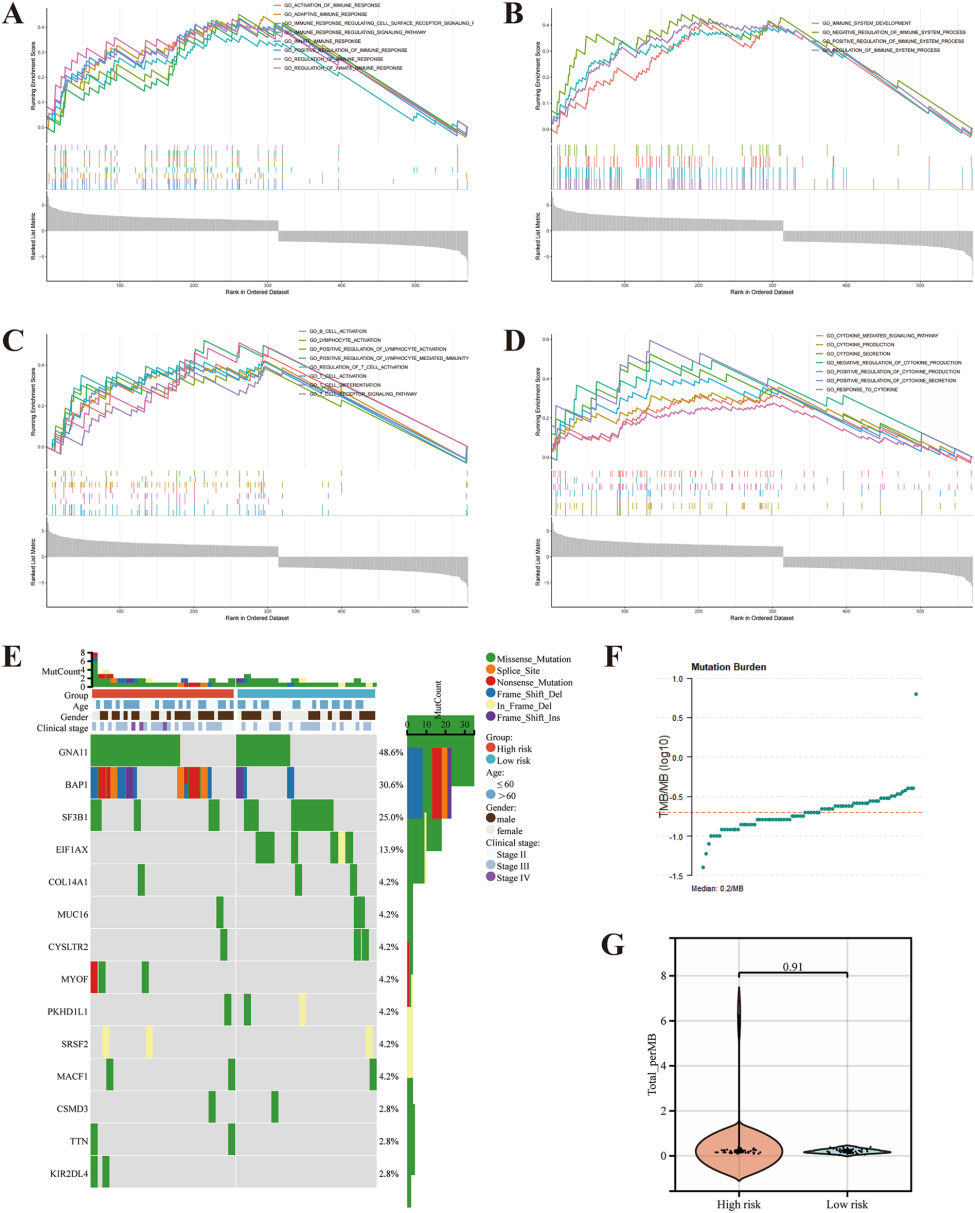
Pathway enrichment analysis and mutation landscape between high- and low-risk patients. Gene Set Enrichment Analysis (GSEA) revealed differentially expressed genes between the two groups of patients, mainly associated with immune response (**A**), immune system process (**B**), immune cell activation (**C**), and cytokine production (**D**). (**E**) The waterfall plot demonstrates the differences in somatic cell mutations between high- and low-risk patients. (**F**) Tumor mutation burden profiles of patients in the TCGA-UVM cohort, with a median of 0.2/MB. (**G**) No statistically significant differences in tumor mutation burden were identified in the high- and low-risk groups.

To explore the genes and biological pathways associated with the prognosis of patients in high- and low-risk groups. 595 DEGs were screened by expression difference analysis, of which 322 DEGs were highly expressed in high-risk patients and 273 DEGs were described in low-risk patients. KEGG and GO analysis revealed that DEGs were mainly associated with cell–cell adhesion, regulation of T-cell activation, and cytokine-cytokine receptor interaction (Fig. 7B, C). The major pathways enriched for DEGs identified through GSEA include immune response, immune system development, immune cell activation, and regulation of cytokine production (Fig. 8A–D).

### Differences in prognostic signature and genomic alterations

We compare the top 15 genes with the highest mutation frequency in the high- and low-risk groups. The mutation frequency of *GNA11* and *BAP1* was higher in the high-risk group, and *SF3B1* and *EIF1AX* were higher in the low-risk group (Fig. 8E). The vast majority of mutations are nonsense mutations. TMB is commonly used as a marker to predict immunotherapy’s efficacy; the TMB ≥ 10 mut/Mb is used as a reference value. The mean TMB value for overall UVM tissue was 0.2 mut/Mb (Fig. 8F). No statistical differences were found in TMB levels compared between the high and low-risk groups (Fig. 8G).

### The relationship between the costimulatory molecules related risk score and drug sensitivity

**Figure 9:**
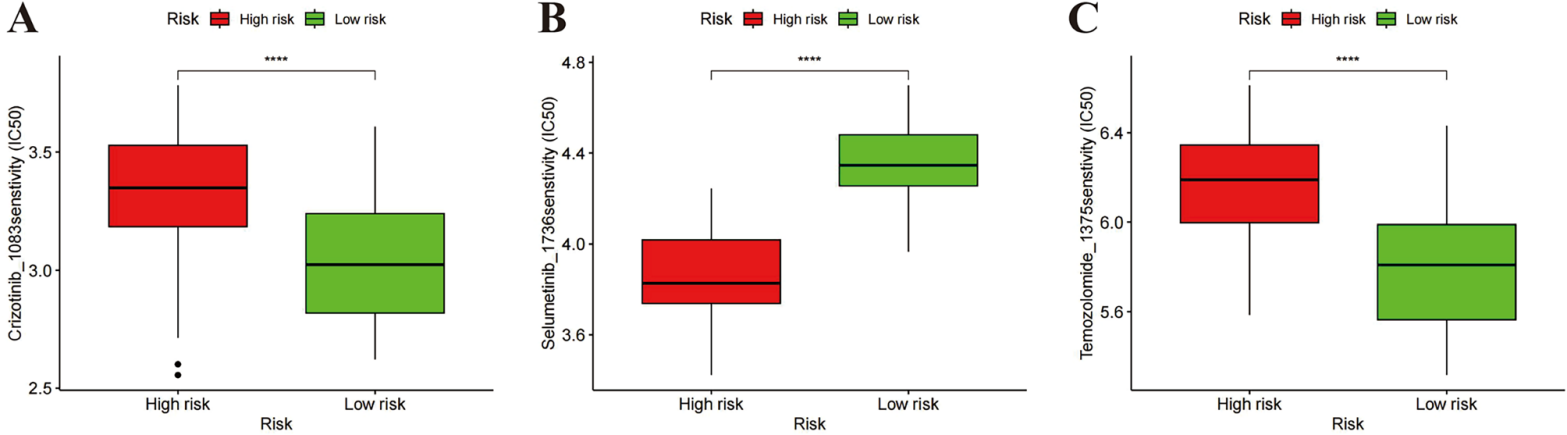
Prediction of chemotherapeutic drug sensitivity. Selecting suitable drugs for patients with OS via OncoPredict. The IC50 value of crizotinib (**A**), selumetinib (**B**) and temozolomide (**C**) in high-risk and low-risk groups of TCGA-UVM. ****P < 0.0001.

In systemic therapy, crizotinib^40^, temozolomide^41^, and selumetinib^42^ have been used alone or in combination to treat mUVM, and chemotherapeutic agents have mostly proved to be ineffective in the treatment of mUVM. We found that high-risk patients may benefit from treatment with crizotinib or temozolomide (Fig. 9A, C), while low-risk patients benefit from treatment with selumetinib (Fig. 9B).

## Discussion

As a malignant tumor, UVM is the most common primary eye tumor in the worldwide, although its incidence is only 5.2 per million in Caucasians^43^. Known risk factors for the development of UVM include light eye color, common cutaneous nevi, propensity to sunburn, iris nevi, and cutaneous freckles^44^. Most patients have a disease limited to the eye at diagnosis, but 40–50% of patients will eventually develop distant metastases^10^. To treat local tumor, iodine 125 brachytherapy has been proven to have the same therapeutic effect as ophthalmologic removal^45,46^. Treatment for the most susceptible liver metastases includes surgical resection, chemoembolization, radioembolization, and percutaneous hepatic infusion^47^. As a clinical treatment challenge, there is no strongly recommended first-line chemotherapeutic drug for the systemic treatment of mUVM^48^. Immune checkpoint inhibitors represented by *PD-1* show very low response rates in mUVM treatment^49^. A clinical study that included nine academic centers found that *PD-1* and *PD-L1* antibodies rarely lead to durable remissions in patients with mUVM^15^. Just in January 2022, tebentafusp a bispecific protein targeting *CD3* and glycoprotein 100, received regulatory approval to conduct international Phase III clinical trials. Previous studies have found that patients treated with tebentafusp reduced the risk of death by 49% and achieved a 1 year survival of 73%^50^. Long-term and relatively intensive follow-up is required, even after complete resection of the primary focus. The selection of the follow-up program is based on the patient’s pathologic stage, as well as genomic sequencing results^1,51^. Transcriptome sequencing can efficiently analyze global gene expression levels, and constructing patient prognostic models through multigene transcript levels has been shown to be meaningful in UVM^52,53^.

CMs are a class of surface membrane proteins involved in T cell activation and proliferation that influence the strength and persistence of the immune response^20^. We accumulated a total of 60 CM genes and extracted the expression of these genes in the TCGA-UVM dataset to screen for prognosis-related genes. LASSO regression analysis selected six CM genes (*TNFRSF19*, *TNFRSF18*, *RELT*, *LTB*, *LTBR*, and *TNFSF13*) as the candidate genes for model construction. Reviewing previous studies, we found that the prognostic impact of CMs has been less studied in UVM. In hepatocellular carcinoma studies, *TNFRSF19* is involved in reshaping the tumor microenvironment, mediating immune evasion^54^. *TNFRSF18* (*GITR*) is highly expressed on Tregs. *GITRL*/*GITR* is involved in inflammation-mediated disease onset and progression primarily through activation of effector T cells and Tregs. Intervention in the *GITRL*/*GITR* pathway may improve the efficacy of antitumor therapy, and anti-GITR biologics are under clinical trials^55^. *LTB* is the β-chain of the heteromeric complex of surface lymphotoxin (*LT*), which binds to the *LT-β* receptor (*LTBR*), and ligand-receptor binding between the two activates NF-κB, affects the inflammatory microenvironment of tumors, and correlates with tumor progression^56^. *LTB* and *LTBR* modulate inflammation and response to immunotherapy by regulating downstream NF-κB signaling and can be used as potential biomarkers for future clinical trials^56,57^. *RELT* was associated with elevated multiple immune checkpoints and infiltration of Treg and myeloid-derived suppressor cells across cancer^58^, also found in this study (Fig. 6D). *TNFSF13*, or *APRIL*, is positively correlated with infiltrating immune cells and stromal cells in the glioma microenvironment^57^. *TNFSF13* is involved in immunosuppression through multiple immunomodulatory pathways and is associated with other immune checkpoints and inflammatory factors^59^. By illustrating the correlation of these five CM genes with tumor progression and the immune microenvironment, we hoped to predict the prognosis of UVM patients by constructing a transcriptome signature.

Our CMs-related signature was highly efficient in predicting the short- and long-term survival and occurrence of metastasis in UVM patients. Through univariate and multivariate Cox regression analyses, we found that risk score was the only risk factor associated with prognosis compared to other factors. Furthermore, we compared the differences in the immune microenvironment between the high and low-risk groups. Patients in the high-risk group had significantly higher immune scores and stromal scores than low-risk patients. In the tumor microenvironment, high-risk patients have a greater infiltration of immune cells, which include activated B cells, activated CD4 T cells, activated CD8 T cells, memory CD4 T cells, memory CD8 T cells, gamma delta T cell, mast cell, MDSC, natural killer T cell, neutrophil, and type 1 helper cell. Pearson correlation analysis revealed that the risk score was positively correlated with the infiltration of multiple immune cells, which include activated CD4 T cell, central CD4 T cell, gamma delta T cell, activated CD8 T cell, central memory CD8 T cell, and CD56 bright natural killer cell. In contrast to other tumors, lymphocyte infiltration in UVM was associated with poor prognosis. Previous studies have found that more stromal cells, T cells CD8 + , NK cells, and macrophage infiltration in the immune microenvironment of UVM were associated with poor prognosis^60^. A study integrating the genome and transcriptome subgrouped 64 UVM patients, in which the subgroup with high immune cell infiltration presented a worse prognosis^61,62^. Although patients in the high-risk group had more immune checkpoint expression, they ultimately had a poor prognosis. The main reason is that UVM is considered an immune-cold tumor due to its low TMB. The average TMB mutation level in TCGA-UVM patients was only 0.2 mut/Mb^63^, substantially lower than the reference value of > 10 mut/Mb. Another interesting view is that the primary lesions of UVM show immunosuppression, and the infiltrating lymphocytes fail to achieve immune defense and promote tumor growth through the production of inflammatory factors^64,65^. Further single-cell and spatial transcriptomic studies are required to further understand the uveal melanoma immune microenvironment.

Through screening DEGs for GO and KEGG analysis, we found significant functional differences between high and low-risk groups, with multiple biological processes related to T-cell activation and cell–cell adhesion regulation. The main biological function of CMs is to activate T cells and participate in producing effector T cells and memory T cells. Abnormal function of cell-to-cell adhesion is closely related to tumor infiltration and metastasis^66^. Tumor cells achieve tumor infiltration by secreting various proteolytic enzymes that disrupt cell–cell adhesion^67^. In addition, cell adhesion molecules are involved in regulating NK cell function^68^. Comparing the differences in somatic mutations, we found that the frequency of mutations in *GNA11* and *BAP1* was higher in the high-risk. Recurrent oncogenic mutations and chromosomal copy number variants recognize UVM. Mutually exclusive mutations occur early in the tumor in almost all cases, with a mutation frequency of 49% in *GNA11*. Driver mutations in the Gα proteins *GNA11* activate the MAP kinase and YAP/TAZ oncogenic signaling pathways in UVM^69^. *BAP1* is a well-recognized tumor suppressor gene. Pathogenic mutations in *BAP1* are associated with susceptibility to multiple tumors^70^. It has been previously shown that BAP1 nuclear staining is a surrogate marker for *BAP1* mutations^71^. Most of the *BAP1*-mutated UVM show loss of chromosome 3. The *BAP1* loss follows monosomy 3 UVM occurrence, although this is not the case for disomy 3 UVM^6,72^. No difference was found by comparing the TMB in the high- and low-risk groups, suggesting that our prognostic model is more sensitive than TMB. Finally, based on the CMs prognostic model with transcriptomic data, we predicted the efficacy of the chemotherapeutic agents that have been used for systemic treatment in UVM. In previous studies, temozolomide was not beneficial in metastatic uveal melanoma, but in our study we found that patients at high risk may benefit in treatment^73^. Crizotinib^74^, a c-Met inhibitor, was proven to block metastasis in a metastatic uveal melanoma model. Successful treatment of a patient with uveal melanoma with crizotinib protected the retina and vision reported in a clinical case report^75^. Transcriptome-based drug sensitivity analysis can provide new ideas for clinical research, but more rigorous clinical validation is needed to truly achieve application.

Our study has limitations. Firstly, although UVM is the most common primary eye tumor, the global incidence is not high, so the number of cases that could be included was limited. Currently, the UVM dataset that can be included is only for primary lesions, and there is a shortage of transcriptomic data for mUVM. Whether our predictive model remains applicable to the metastasis dataset in the future is unknown. Second, this study did not explore the functions of the candidate genes in the CM signature in UVM because limited research was conducted. Finally, high infiltration of immune cells in the immune microenvironment of UVM is associated with poor prognosis, and the exact mechanism is still unknown. It is hoped that single-cell and spatial transcriptome sequencing can be used in subsequent studies to reveal the immune landscape within and around the UVM tumor.

## Conclusions

In our study, we elucidated the differential expression of CM genes in UVM patients for the first time. The CMs-related signature can effectively identify high-risk subgroups with poor prognosis. Our model elaborated that poor prognosis in high-risk patients may be associated with high levels of immune cell infiltration. Therefore, we believe that larger-scale cases can validate our signature and guide the treatment and prognosis of UVM patients.

### Supplementary Information

Supplementary Table 1.

Supplementary Table 2.

## Data Availability

Genomic data, transcriptomic data, and clinical information of UVM in this study are available from the TCGA and GEO datasets.

## Abbreviations

APC: Antigen-presenting cells
AUC: Area under curve
CDF: Cumulative distribution function
CM: Costimulatory molecule
DEG: Differentially expressed gene
GDSC2: Genomics of drug sensitivity in cancer 2
GEO: Gene expression omnibus
GO: Gene ontology
GSEA: Gene set enrichment analysis
IC50: Half maximal inhibitory concentration
ICI: Immune checkpoint inhibitor
KEGG: Kyoto Encyclopedia of Genes and Genomes
K-M: Kaplan–Meier
LASSO: Least absolute shrinkage and selection operator
MHC: Major histocompatibility complex
mUVM: Metastatic uveal melanoma
OS: Overall survival
PCA: Principal component analysis
PFI: Progression-free interval
ROC: Receiver operating characteristic
TCGA: The cancer genome atlas
TIL: Tumor-infiltrating lymphocyte
TIME: Tumor immune microenvironment
timeROC: Time-dependent receiver operating characteristic curve
TMB: Tumor mutation burden
UVM: Uveal melanoma
