# Supplementary material for: Identification and validation of a costimulatory molecule-related signature to predict the prognosis for uveal melanoma patients
Source: Sci Rep. 2024 Apr 21;14:9146. doi: 10.1038/s41598-024-59827-5 (PMC11033288; doi:10.1038/s41598-024-59827-5)
Supplement: Supplementary file 2 — Supplementary Table 2. [file 41598_2024_59827_MOESM2_ESM.docx]

| **Variables** | **Validation cohort**  **(n=63)** | **Risk score^a^** | | ***P*-value** |
| --- | --- | --- | --- | --- |
|  |  | **High risk** | **Low risk** |  |
| **Gender** |  |  |  | 0.674 |
| Female | 24 | 11 | 13 |  |
| Male | 39 | 20 | 19 |  |
| **Age (mean±SD, years)** | 61.00±12.28 | 62.99±11.93 | 59.07±12.50 | 0.055 |
| ≤ 60 years | 28 | 10 | 18 |  |
| > 60 years | 35 | 21 | 14 |  |
| **Metastasis** |  |  |  | ***0.003**** |
| Yes | 35 | 23 | 12 |  |
| No | 28 | 8 | 20 |  |
| **Monosomy 3** |  |  |  | NA |
| Yes | 32 | NA | NA |  |
| No | 18 | NA | NA |  |
| Partial alteration | 5 | NA | NA |  |
| NA | 8 | NA | NA |  |

**Supplementary Table 2. Detailed clinical information of GSE22138 patients**

^a^Risk scores were calculated by multiplying the expression level of the genes and the multivariate Cox proportional hazards coefficient. Patients were divided into high- and low-risk groups based on the median risk score.

*Font bold indicates statistical significance (p < 0.05).

Abbreviations: NA, not applicable.
